# A fuzzy feature fusion method for auto-segmentation of gliomas with multi-modality diffusion and perfusion magnetic resonance images in radiotherapy

**DOI:** 10.1038/s41598-018-21678-2

**Published:** 2018-02-19

**Authors:** Lu Guo, Ping Wang, Ranran Sun, Chengwen Yang, Ning Zhang, Yu Guo, Yuanming Feng

**Affiliations:** 1 Department of Biomedical Engineering, Tianjin University, Tianjin, 300072 China; 2 Department of Radiation Oncology, Tianjin Medical University Cancer Institute & Hospital, Tianjin, 300060 China; 3 East Carolina University, Greenville, NC 27834 USA

## Abstract

The diffusion and perfusion magnetic resonance (MR) images can provide functional information about tumour and enable more sensitive detection of the tumour extent. We aimed to develop a fuzzy feature fusion method for auto-segmentation of gliomas in radiotherapy planning using multi-parametric functional MR images including apparent diffusion coefficient (ADC), fractional anisotropy (FA) and relative cerebral blood volume (rCBV). For each functional modality, one histogram-based fuzzy model was created to transform image volume into a fuzzy feature space. Based on the fuzzy fusion result of the three fuzzy feature spaces, regions with high possibility belonging to tumour were generated automatically. The auto-segmentations of tumour in structural MR images were added in final auto-segmented gross tumour volume (GTV). For evaluation, one radiation oncologist delineated GTVs for nine patients with all modalities. Comparisons between manually delineated and auto-segmented GTVs showed that, the mean volume difference was 8.69% (±5.62%); the mean Dice’s similarity coefficient (DSC) was 0.88 (±0.02); the mean sensitivity and specificity of auto-segmentation was 0.87 (±0.04) and 0.98 (±0.01) respectively. High accuracy and efficiency can be achieved with the new method, which shows potential of utilizing functional multi-parametric MR images for target definition in precision radiation treatment planning for patients with gliomas.

## Introduction

To avoid missing target and spare critical healthy brain tissue around the target volume in radiotherapy treatment planning of gliomas, cancerous tissue involvement must be correctly defined in the gross tumour volume (GTV) delineation. Conventional T1-weighted contrast-enhanced (T1C) and T2-weighted (T2) magnetic resonance imaging (MRI) reveal soft tissue with high contrast and could help improve the accuracy of tumour volume definition^1^. However, conventional MRI does not accurately show the actual tumour borders of glial neoplasms because it reflects only anatomic rather than functional properties of the tumour^2^. And tumour cells could be found in serial biopsies beyond signal intensity changes on T2 MRIs^3^. In contrast, advanced imaging techniques, such as diffusion and perfusion MRI, provide functional information about the tumour microenvironment and enable more sensitive detection of the tumour extent. For instance, the parameter of apparent diffusion coefficient (ADC) is derived from diffusion-weighted imaging (DWI) and is proved to be correlated reciprocally with tumour cellularity^4^. Another useful parameter of fractional anisotropy (FA) is calculated from diffusion tensor imaging (DTI) and is sensitive to the changes in fibre bundles. Furthermore, dynamic-susceptibility contrast (DSC) and its derived parameter of relative cerebral blood volume (rCBV) can be used to assess the tumour vascularity.

Those diffusion and perfusion parameters have been investigated to define accurate tumour extent and delineate target volume in radiation treatment planning. For instance, DTI-derived FA values was used to detect white-matter abnormalities resulting from tumour infiltration and reduce the size of the planning-target volume (PTV), resulting in escalated doses without an increase in normal tissue complication probability (NTCP) in radiotherapy treatment planning for patients with high-grade gliomas^5^. Additionally, FA values was also used to identify tumour cell infiltration along white matter tracts in high-grade gliomas in order to preserve coverage of the likely routes of dissemination and spare uninvolved brain, generating a better radiotherapy target volume delineation^6^. Furthermore, the integration of ADC, rCBV and metabolic information of proton MR spectroscopic imaging (^1^H-MRSI) is able to discriminate infiltrating tumour from surrounding vasogenic oedema or normal tissues for improving the evaluation of glioblastoma extent^7,8^.

As the types of MR modalities involved in studies increase, accurate manual delineation of GTV in radiotherapy treatment planning becomes highly time-consuming and complicated for radiation oncologists, which may lead to intra- and inter-observer variability^9–11^. The application of automatic segmentation methods might be helpful to solve this problem. Recently, most of the automatic segmentation methods using information integration of multimodal MR images of patients with gliomas focus on deformable models and clustering techniques^12^. Although methods based on deformable models allow for real-time applications with high computational efficiency, they are sensitive to the initial condition and local functional minimum, which may limit the applications in MR images with inherent noise and vague tumour border^13–15^. Compared with deformable models, clustering techniques such as K-mean and Fuzzy C-means (FCM) algorithms treat structures in medical images as patterns and use techniques from pattern recognition fields to perform segmentation^13^. Thus, clustering techniques have more applications for the medical images where the boundaries of structures or areas of interest are often poorly defined. In a recent study, spatial FCM algorithm was used to improve the accuracy in segmentation of different pathogenic region of glioblastoma multiforme (GBM) in multi-parametric (ADC, rCBV, T2-weighted) MRI fusion framework^16^.

**Figure 1:**
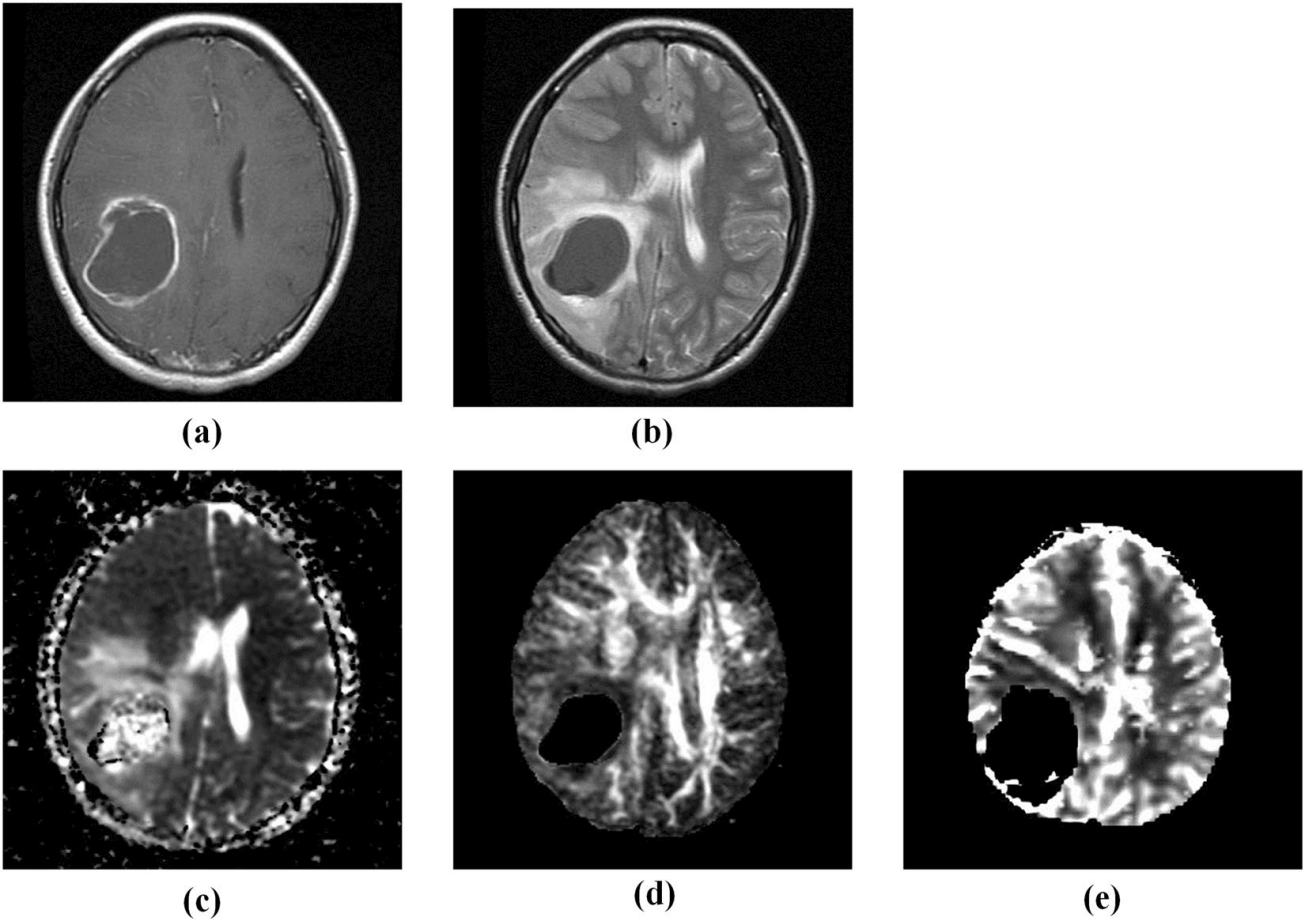
Multimodal images of a patient with glioblastoma multiforme (GBM). (**a**) T1-weighted contrast-enhanced image, (**b**) T2-weighted image, (**c**) apparent diffusion coefficient (ADC) map, (**d**) fractional anisotropy (FA) map, (**e**) relative cerebral blood volume (rCBV) map. The appearances of tumor in anatomical and functional images are different from each other.

**Figure 2:**
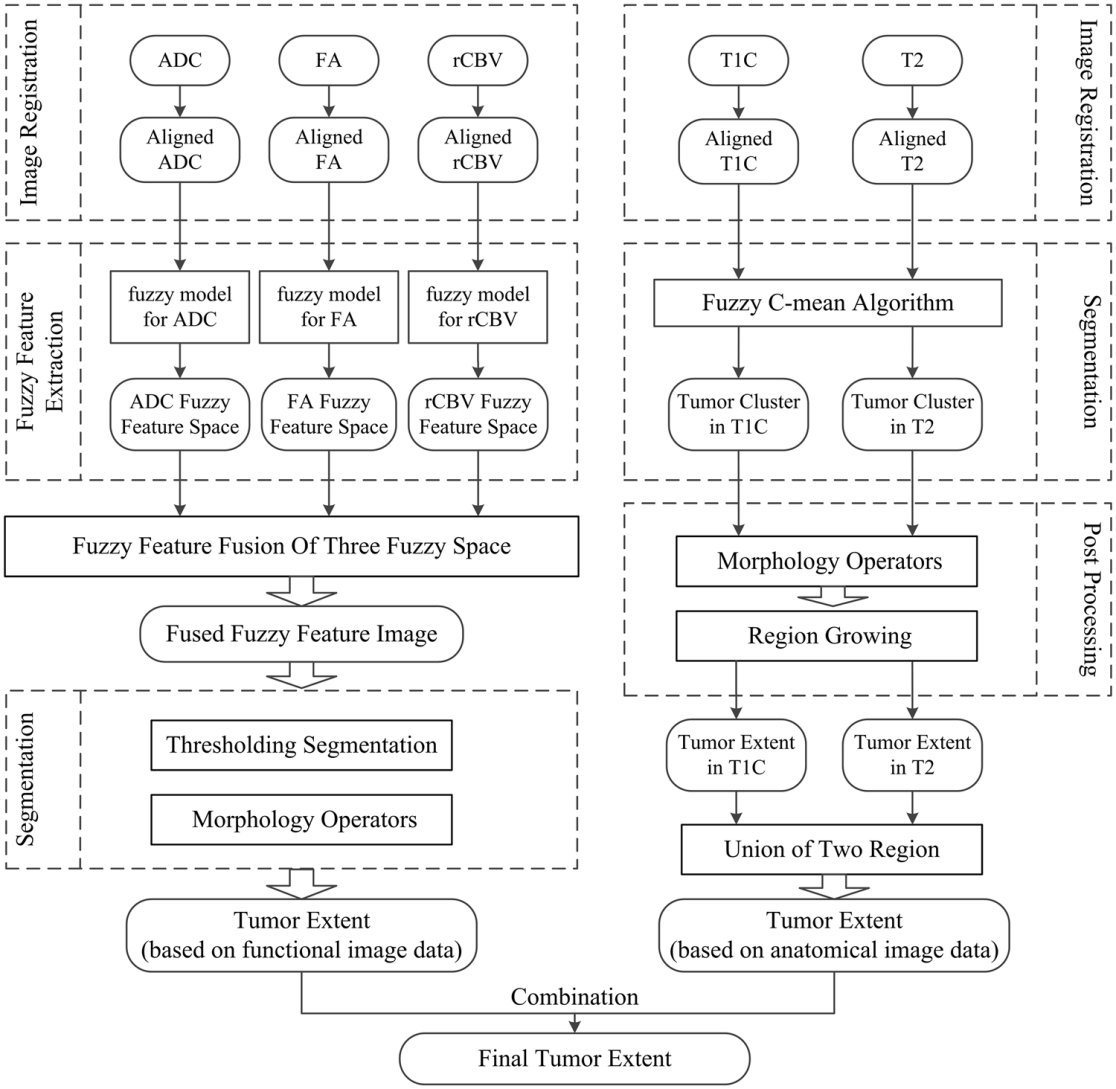
Framework of fuzzy fusion and tumor segmentation using multi-modality diffusion and perfusion MRI images. The original input images are noted MRI sequences of T1-weighted contrast-enhanced images (T1C), T2-weighted images (T2), and parameter maps of apparent diffusion coefficient (ADC), fractional anisotropy (FA) and relative cerebral blood volume (rCBV). Final tumor extent is the combination of segmentation results derived from functional and anatomical parts as shown in the left and right columns.

Nevertheless, accurate automatic segmentation of gliomas in multimodal MR images remains a challenging issue for three main reasons: (1) the tumour heterogeneous nature and the difference in image acquisition method make one tumour shows different shape, size and location in different image modality (Fig. 1); (2) the ambiguous tumour border may deteriorate in the image with decreased resolution, especially in diffusion and perfusion parameter image such as ADC, FA and rCBV; (3) the partial volume effects and the inherent noise in imaging system could produce negative influence to the segmentation results^9^.

To explore solutions for the problems mentioned above, a fuzzy feature fusion method using multi-modality diffusion and perfusion MR images (ADC/FA/rCBV) is presented in this study for automatic segmentation of gliomas in radiation treatment planning. The new method provides each voxel with a possibility value belonging to the tumour which helps in preventing missing high risk regions in GTV definition in radiation treatment planning.

## Methods and Material

In this study, all methods were carried out in accordance with relevant guidelines and regulations. All images were collected and used with the approval of the Ethics Committee of Tianjin University Biomedical Engineering and patients’ written consents. And informed consents were obtained from all of the patients.

### Fuzzy feature fusion method

In assessing gliomas with functional multi-parametric images, commonly used descriptions about the appearance of gliomas in images by clinical radiation oncologists were considered. In ADC maps, the majority of image intensity attributable to tumour, necrosis and oedema in peritumoral area are higher than normal-appearing white matter^17^. And the FA values would decrease in case of tumour infiltration because the reduction of the directional order of axonal tracts^18^. For rCBV values, they would increase in regions adjacent to the tumour because the tumour growth and invasion is dependent on developing blood supply^19^. Therefore, a glial tumour often presents as a relative bright region (hyper-signal) in ADC and rCBV map, while a dark region (hypo-signal) in FA map. However, these descriptions are very fuzzy and there is no commonly accepted threshold standard of these parametric values for precisely defining the tumour border. Thus, we considered transforming these fuzzy linguistic descriptions into mathematics fuzzy models and treating the definition of tumour extent as fuzzy image processing which understands and represents image features as fuzzy set^20^. For each modality (ADC/FA/rCBV), we created one mathematics fuzzy model that transforms the image volume into fuzzy feature space in which the fuzzy feature represents the possibility belonging to tumour^9^. Based on the fuzzy fusion result of the three fuzzy feature spaces, the auto-segmentation was conducted to generate the final result on functional multi-parametric image data.

Additionally, conventional anatomical MRI image data (T1C and T2) was also included to improve the accuracy and reliability of final segmentation. FCM algorithm was used in the tumour segmentation in anatomical MRI image data.

The framework of fuzzy fusion and tumour segmentation is shown in Fig. 2. In this framework, functional multi-parametric images and anatomical images are processed separately, and the final segmentation result is the combination of segmentation derived from functional and anatomical parts.

#### Tumour extent definition in functional multi-parametric images

Fuzzy feature extraction: In the application of fuzzy set theory, estimation of membership functions from image data is an important step^21^. In this study, the membership functions for each parametric value are fuzzy models for extracting fuzzy feature that represents the possibility of each voxel belonging to tumour. There are many methods for generating membership functions such as perception-based method^22^, heuristic methods^23^ and histogram-based methods^24^. For taking full advantage of the statistical probability distributions of multi-parametric values of tumour in functional maps, one histogram-based method was developed in the generation of membership functions which consists of following two steps.

### Sample collection

In order to avoid bias in sample collection conducted by a single institution, parametric values of tumour from our clinical multi-parametric MRI datasets and related research articles published by different institution were treated as samples for each modality. The clinical image datasets were obtained from 4 patients with biopsy-confirmed gliomas including 2 low-grade and 2 high-grade astrocytomas. Each patient underwent DWI, DTI and DSC imaging, the ADC, FA, rCBV maps were calculated respectively. The ADC values in hyperintense region such as solid enhancing tumour mass, tumour margin, infiltrating tumour, tumour-infiltrated oedema and infiltrated tissue are considered as reasonable representation of tumour. 16 samples (ADC mean value ± standard deviation) were generated from the 4 clinical image datasets (ADC maps) in which the ADC values were calculated in regions of interest (ROIs) with size of 25 pixels placed in non-cystic mass, tumour margins, areas of abnormal MR signal surrounding the enhancing tumours. In addition, 16 ADC values from 5 published articles^7,8,25–27^ were added as samples, making that the total sample size for ADC values 32. The FA values in hypointense region such as solid enhancing tumour mass and tumour-infiltrated tissue can be used to generate samples of tumour^18^. On FA maps in our clinical datasets of 4 patients, 16 FA values (mean value ± standard deviation) were measured by placing ROIs of 25 pixels in enhancing tumour and regions adjacent to enhancing tumour. Additionally, 5 FA values were generated in similar locations from 3 published articles^25,26,28^, resulting in that the total sample size for FA values 21. Because the rCBV values in and adjacent to tumour are often higher than in the corresponding contralateral areas, rCBV ratio values = rCBV [tumour]/rCBV [contralateral normal white matter] is usually used in evaluation^8^. 12 rCBV ratio values (mean value ± standard deviation) were generated from our clinical datasets of 3 patients, in which the rCBV values were calculated in ROIs placed in solid enhancing tumour, hyperintense non-enhancing peritumoral area and contralateral normal white matter. Moreover, 20 rCBV ratio values were measured in similar locations on rCBV maps from 5 published articles^7,8,26,27,29^. Thus, the sample size for rCBV ratio value was 32.

### Fuzzy model creation

**Figure 3:**
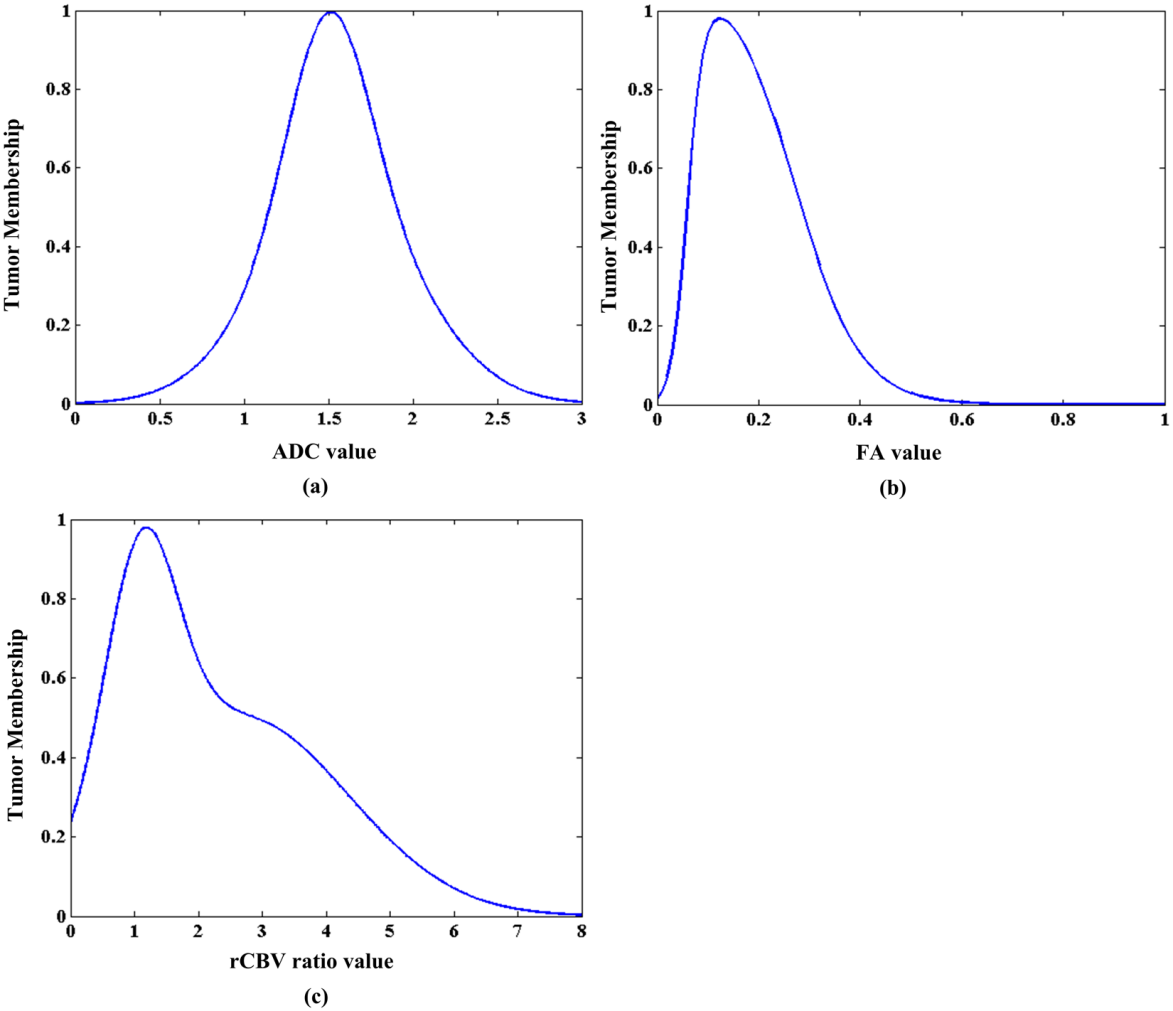
The membership functions of the tumorous tissue in multi-parametric MRI images. (**a**) Membership function in apparent diffusion coefficient map (ADC) noted as $M{F}_{ADC}({\nu }_{ADC})$, (**b**) membership function in fractional anisotropy map (FA) noted as $M{F}_{FA}({\nu }_{FA})$, (**c**) membership function in relative cerebral blood volume map (rCBV) noted as $M{F}_{CBV}({\nu }_{CBV})$.

**Figure 4:**
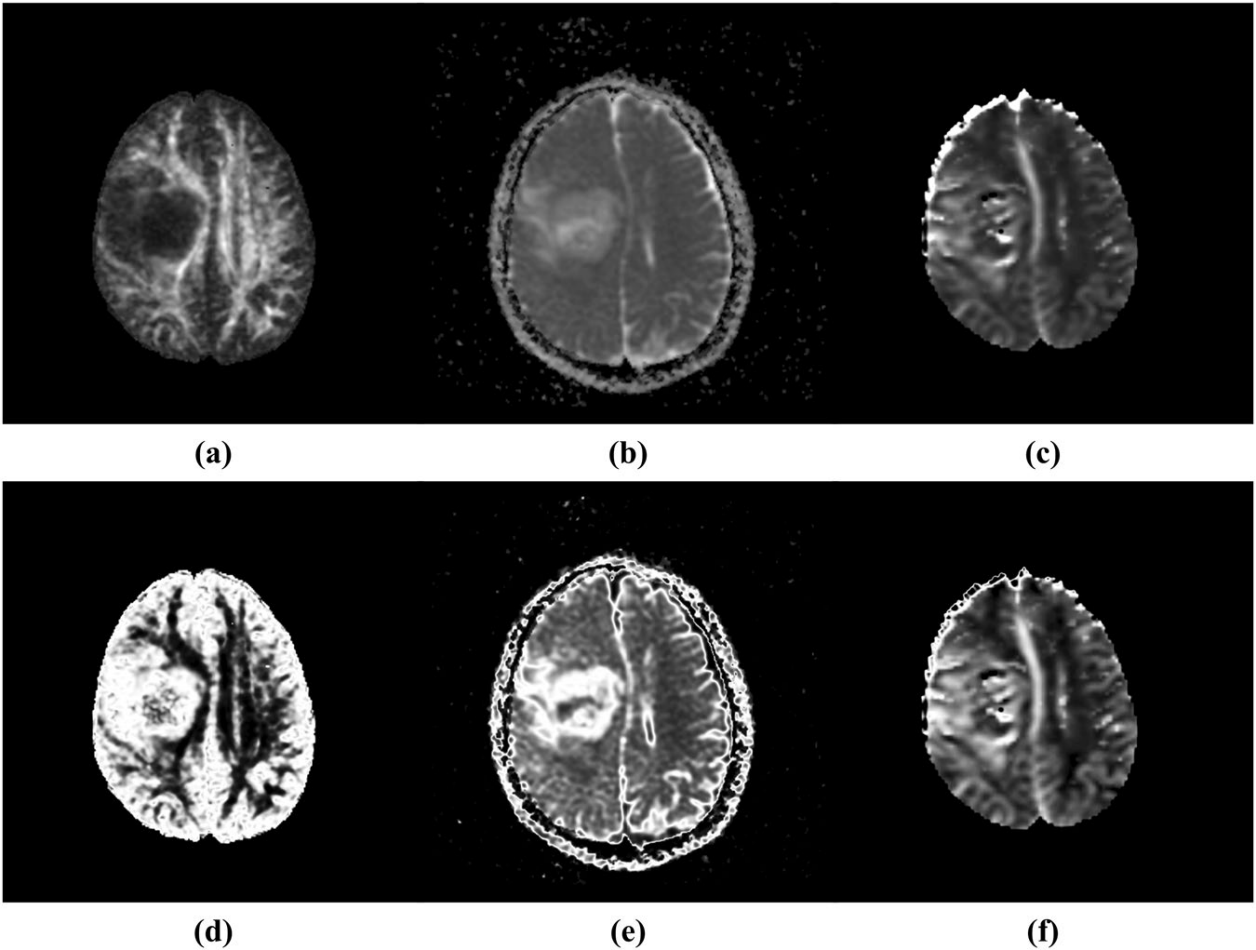
One slice of three parametric MRI image volumes and the corresponding three fuzzy feature spaces, in which voxel values represent the probability belonging to tumour. (**a**) Fractional anisotropy map (FA), (**b**) apparent diffusion coefficient map (ADC), (**c**) relative cerebral blood volume map (rCBV), (**d**) fuzzy feature space of FA map noted as $F{S}_{FA}$, (**e**) fuzzy feature space of ADC map noted as $F{S}_{ADC}$, (**f**) fuzzy feature space of rCBV map noted as $F{S}_{CBV}$.

Fuzzy statistics method was used to calculate the tumour membership frequency of parametric values using the samples of each modality as mentioned above^30^. The tumour membership frequency of each fuzzy interval of parametric value was represented using histograms and treated as probability distributions of membership functions for parametric values. Then, the histograms were modelled with a mixture of parameterized functions to generate the membership function. Curve fitting was applied in the definition of the most suitable mixture format of parameterized functions with low fitting error. The membership functions corresponding to ADC, FA and rCBV values are defined with following equations:

1 $$M{F}_{ADC}({\nu }_{ADC})=0.5\times {e}^{-{(\frac{{\nu }_{ADC}-1.5}{0.33})}^{2}}+0{\rm{.5}}\times {e}^{-{(\frac{{\nu }_{ADC}-1.57}{0.66})}^{2}}$$

2 $$M{F}_{FA}({\nu }_{FA})=1.1\times (\frac{1}{1+{e}^{-60({\nu }_{FA}-0.06)}}-\frac{1}{1+{e}^{-15.76({\nu }_{FA}-0.27)}})$$

3 $$M{F}_{CBV}({\nu }_{CBV})=0.65\times {e}^{-{(\frac{{\nu }_{CBV}-1.1}{0.8})}^{2}}+0{\rm{.5}}\times {e}^{-{(\frac{{\nu }_{CBV}-2.68}{2.37})}^{2}}$$

where ${\nu }_{ADC}$, ${\nu }_{FA}$ and ${\nu }_{CBV}$ represents the parametric value of voxel in ADC, FA and rCBV map respectively and $M{F}_{ADC}({\nu }_{ADC})$, $M{F}_{FA}({\nu }_{FA})$ and $M{F}_{CBV}({\nu }_{CBV})$ represents the membership function corresponding to ADC, FA and rCBV ratio values respectively. The proposed membership functions of the tumorous tissue in multi-parametric MRI images are shown in Fig. 3. A membership function maps each voxel of image volume to a membership value between 0 and 1 that represents the possibility belonging to tumour. Thus, the membership functions, $M{F}_{ADC}({\nu }_{ADC})$, $M{F}_{FA}({\nu }_{FA})$ and $M{F}_{CBV}({\nu }_{CBV})$, are fuzzy models that transform the three parametric MRI image volumes into three fuzzy feature spaces, noted as $F{S}_{ADC}$, $F{S}_{FA}$ and $F{S}_{CBV}$, in which the voxel values represent the possibility belonging to tumour (Fig. 4).

Fuzzy fusion of three fuzzy feature spaces: Isolated diffusion/perfusion parameters only reveal partial information about brain-tumour microenvironments and are insufficient to enable clinicians make crucial decisions throughout the whole treatment. To take advantage of multimodal fuzzy information and compromise the three memberships, the geometric mean operator was used in this study for fuzzy fusion of the three fuzzy feature spaces^9^. The fusion process was conducted on voxel level to generate each voxel value in fused fuzzy feature volume noted as $F{S}_{F{\rm{u}}sion}$ which is defined as following:

4 $$F{S}_{F{\rm{u}}sion}(v)=\sqrt[3]{F{S}_{ADC}(v)F{S}_{FA}(v)F{S}_{CBV}(v)}$$

where $v$ represents the voxel in fused fuzzy feature volume, and $F{S}_{ADC}(v)$, $F{S}_{FA}(v)$ and $F{S}_{CBV}(v)$ represents the membership value of the corresponding voxel in fuzzy feature space of ADC, FA and rCBV ratio values respectively.

**Figure 5:**
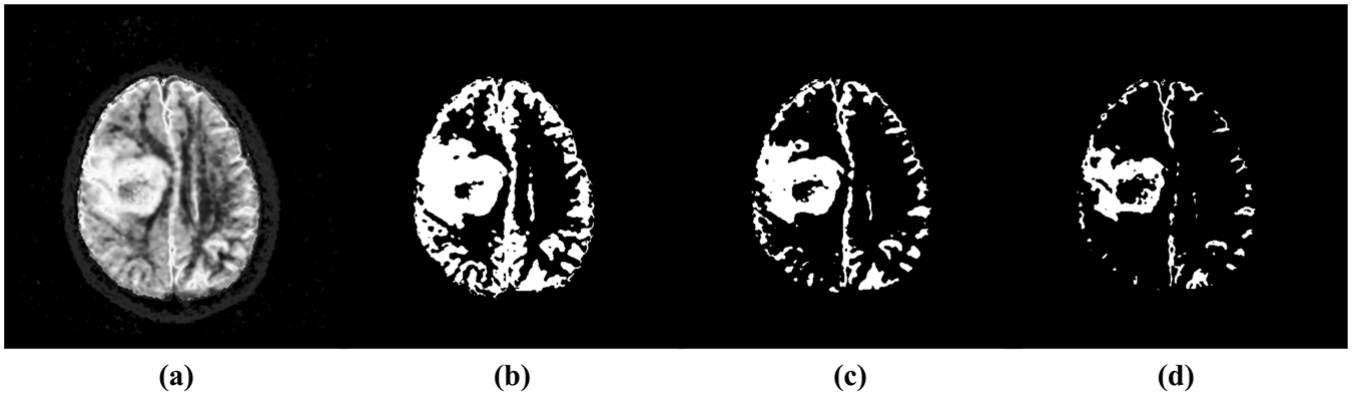
The fused fuzzy feature volume (**a**) and regions with different memberships. (**b**) ≥0.6, (**c**) ≥0.7, (**d**) ≥0.8.

Segmentation after fusion: To extract a region containing voxels with high possibility belonging to tumour, segmentation was applied on $F{S}_{F{\rm{u}}sion}$. As shown in Fig. 5, the regions that consist of voxels with membership $F{S}_{F{\rm{u}}sion}(v)$ ≥ 0.6 cover most of the abnormal area in $F{S}_{F{\rm{u}}sion}$ as compared with the contralateral normal area. However, there are still some normal voxels that are included in the regions with membership $F{S}_{F{\rm{u}}sion}(v)$ ≥ 0.6. Thus, one clinical radiation oncologist evaluated the $F{S}_{F{\rm{u}}sion}$ images and manually limited the range of thresholding segmentation the area with high possibility belonging to tumour. The region with membership $F{S}_{F{\rm{u}}sion}(v)$ ≥ 0.6 was extracted in the limited area. Subsequently, a group of morphology operators were applied to each image slice in this region as following: filling holes with two-dimensional four-connected neighbourhood, eroding with a flat disk-shaped structuring element with radius of 3-pixels twice, dilating twice with the same structuring element as eroding, generating the maximum region with 8-connectivity. The generated region after morphology operators served as the tumour volume that was segmented in multi-parametric functional data sets and was noted as $Functio{n}_{auto}$.

#### Tumour extent definition in anatomical MR images

FCM algorithm was applied for segmenting tumour in T1C and T2 MRI image data. The gliomas often show a contrast-enhanced region in T1C image which is much bright than normal-appearing peritumoral white matter and other brain tissues such as grey matter and cerebrospinal fluid (CSF). Thus, T1C images were classified into three clusters, including solid enhancing tumour, normal brain tissues and non-brain image pixels. In T2 images, the hyperintense area often reveals tumour existence because of the tumour-infiltrated oedema of gliomas. This area is bright than normal peritumoral brain tissues. Therefore, three clusters were classified in T2 images which include tumour region, normal brain tissues and non-brain image pixels.

Some morphological processing operators were used to fill small holes and disconnect the tumour region with normal tissues in the same cluster. Afterwards, to merge adjacent pixels belonging to the tumour cluster, the region growing algorithm was applied with manually selected seed in the centre of tumour area. The union of region growing results in T1C and T2 images was used as the tumour extent definition in anatomical MR images and noted as $Anatom{y}_{auto}$.

#### Combination of anatomical and functional information

The auto-segmentation results from anatomical and functional image data were used to generate the final auto-segmentation result of tumour volume which was formulated as following:

5 $$GT{V}_{auto}=Anatom{y}_{auto}\cup Functio{n}_{auto}$$

where $GT{V}_{auto}$ represents the final auto-segmented gross tumour volume which is the union of $Anatom{y}_{auto}$ and $Functio{n}_{auto}$.

#### Evaluation

Imaging and preprocessing: Images for evaluation were acquired from 9 patients who were histopathologically diagnosed with gliomas (6 high-grade and 3 low-grade). The MR images were acquired for each patient on a 1.5 T MR scanner (GE Signa Excite), including T1C, T2, DWI, DTI and perfusion weighted (PWI) images. Whole-brain axial T2 images were acquired using fast spin echo sequence with TR/TE = 3900/92 ms, image matrix = 512 × 512, field of view = 240 × 240 mm^2^ and slice thickness = 5 mm; DWI images were acquired using spin-echo echo-planar sequence with TR/TE = 4900/85 ms, image matrix = 256 × 256, field of view = 240 × 240 mm^2^, slice thickness = 6 mm, number of slices = 30 and b-values of 0 and 1000 s/mm^2^ in three orthogonal directions; DTI images were acquired using spin-echo echo-planar sequence with TR/TE = 6000/85 ms, image matrix = 256 × 256, field of view = 240 × 240 mm^2^, slice thickness = 6 mm, number of slices = 19, diffusion-sensitizing gradient encoding applied in 25 directions with b value of 1000 s/mm^2^, and one image set acquired without diffusion-sensitizing gradients; PWI images were acquired by a gradient-echo echo-planar imaging sequence with TR/TE = 2000/40 ms, flip angle = 90°, image matrix = 128 × 128, field of view = 240 × 240 mm^2^, slice thickness = 6 mm, number of measurements = 60 and number of slices = 17. After the first 10 acquisitions, a bolus of gadopentetate dimeglumine (0.2 mmol/kg) was injected as the contrast agent with a flow rate of 3 ml/s, followed by a 20 ml saline flush. Finally an axial T1C imaging sequence was acquired using spin echo sequence with TR/TE = 520/14 ms, image matrix = 512 × 512, field of view = 240 × 240 mm^2^ and slice thickness = 5 mm.

Multi-parametric images were generated in a GE medical imaging workstation (FuncTool 9.4.05a, GE Signa Excite) by analysing the original DWI, DTI and PWI image sets. The ADC and FA maps were generated from DWI and DTI images on a voxel-level basis respectively after calculating the eigenvalues of diffusion tensor in each voxel. According to the indicator dilution theory for intravascular tracers, voxel-based rCBV maps were created by integrating the area under the concentration-time curve of the contrast agent with the measurement of arterial input function (AIF) to position the ROI on the most visible large contralateral arterial vessel.

The 3D dual-modality rigid-body registrations were completed using the automatic image registration tool of MIM 5.2 (MIM Software Inc., Cleveland, OH). Based on the mutual information measure, the software mapped the secondary image sets of T2, ADC, FA and rCBV to the primary image set of T1C respectively.

### Delineation of ground truth

For evaluating the performance of the proposed framework based on fuzzy feature fusion method for auto-segmentation of gliomas, the GTV delineation on the same MR images was performed by one experienced radiation oncologist who was blinded to the auto-segmentation results. All GTV delineation results were reviewed and validated by another experienced radiation oncologist to minimize uncertainty and assure accuracy of the delineation. For each patient, GTVs were contoured on anatomical and functional multi-parametric MR image data respectively and combined to generate final result. On T1C and T2 images, the contrast-enhanced and hyperintense region was outlined and noted as $T1{C}_{manual}$ and $T{2}_{manual}$ respectively^31^. The combination result of the two regions was used as GTV on anatomical MR images and noted as $Anatom{y}_{manual}=T1{C}_{manual}\cup T{2}_{manual}$. According to the ADC values of tumour-infiltrated oedema and tumour margin shown in a published article, the hyperintense regions with ADC value higher than 1.24 × 10^−3^ mm^2^/s and 1.11 × 10^−3^ mm^2^/s were delineated for high-grade and low-grade gliomas respectively and noted as $AD{C}_{manual}$^31^. The delineation process was applied by comparing with contralateral normal brain tissue, and excluding the normal hyperintense region in ADC map such as CSF. For FA maps, compared with the contralateral normal white matter, the hypointense regions with FA value lower than 0.286 and 0.17 were contoured for high-grade and low-grade gliomas respectively and noted as $F{A}_{manual}$. The thresholding FA values used for tumour-infiltrated oedema and intratumoral region were the same as in a published article^32^. Before delineation on rCBV maps, rCBV ratio values = rCBV[tumour]/rCBV[contralateral normal white matter] were calculated for each voxel in and surrounding the contrast-enhanced tumour area shown in T1C and T2 images. The rCBV[contralateral normal white matter] was obtained by calculating the mean rCBV value in a ROI of 25 pixels placed in the contralateral and symmetrical normal white matter. Subsequently, in accordance with the rCBV ratio values of tumour mass shown in a published study, hyperintense regions with rCBV ratio higher than 5.18 and 2.32 were delineated for high-grade and low-grade gliomas respectively and noted as $CB{V}_{manual}$^33^. The necrosis, cystic mass, large vessels and haemorrhage were excluded by referring to the T1C and T2 images. Based on the clinical experience of the observer, the GTV on functional multi-parametric MR image was obtained by integrating the three regions and noted as $Functio{n}_{manual}=combination(AD{C}_{manual},F{A}_{manual},CB{V}_{manual})$. Finally, the manually delineated GTV was generated by the union of anatomical and functional results and noted as $GT{V}_{manual}=Anatom{y}_{manual}\cup Functio{n}_{manual}$.

### Quantitative evaluation

The manual delineation results were used as the ground truth. Then, the quantitatively comparisons between manually delineated GTV ($GT{V}_{manual}$) and automatically segmented GTV ($GT{V}_{auto}$) in terms of volume and shape were assessed for each patient^11^. Additionally, sensitivity and specificity were calculated to evaluate the performance of auto-segmentation.

The volume difference between $GT{V}_{auto}$ and $GT{V}_{manual}$ was calculated as following^11^:

6 $${\rm{\Delta }}V( \% )=\frac{|{V}_{auto}-{V}_{manual}|}{{V}_{manual}}\times 100$$

where ${V}_{auto}$ is the volume of $GT{V}_{auto}$ and ${V}_{manual}$ is the volume of $GT{V}_{manual}$. For comparison of the shape of two volumes, Dice’s similarity coefficient (DSC) was used to evaluate the concordance between $GT{V}_{auto}$ and $GT{V}_{manual}$, which is described as^34^:

7 $$DSC=\frac{2(GT{V}_{auto}\cap GT{V}_{manual})}{(GT{V}_{auto}+GT{V}_{manual})}$$

Apparently, DSC values range from 0 to 1, in which 0 means total disunity of two volumes and 1 means the two volumes are equal and total unity in shape. The evaluations of sensitivity and specificity were conducted as following:

8 $$Sensitivity=\frac{{V}_{turepositive}}{{V}_{turepositive}+{V}_{faslenegative}}$$

9 $$Specificity=\frac{{V}_{turenegative}}{{V}_{turenegative}+{V}_{falsepositive}}$$

where the ${V}_{turepositive}$ was the intersection of $GT{V}_{auto}$ and $GT{V}_{manual}$, ${V}_{faslenegative}$ was the volume in $GT{V}_{manual}$ but not included in $GT{V}_{auto}$, ${V}_{turenegative}$ was the volume not included in both of $GT{V}_{auto}$ and $GT{V}_{manual}$ and ${V}_{falsepositive}$ was the over-segmented volume in $GT{V}_{auto}$ but not included in $GT{V}_{manual}$^11^.

## Result

The comparison of volumes between manually delineated GTV and automatically segmented GTV is summarized in Table 1. It can be seen that the volumes of GTVs defined by automatic segmentation differs from the volumes of GTVs defined by manual delineation on each patient. ${\rm{\Delta }}V$ ranges from 1.66% (patient No. 9) to 19.59% (patient No. 1) with the mean value of 8.69% (±5.62%).

**Table 1:** Comparison of GTV volumes defined by manual delineation ($GT{V}_{manual}$) and automatic segmentation based on fuzzy feature fusion method ($GT{V}_{auto}$).

Patient	${\boldsymbol{G}}{\boldsymbol{T}}{{\boldsymbol{V}}}_{{\boldsymbol{m}}{\boldsymbol{a}}{\boldsymbol{n}}{\boldsymbol{u}}{\boldsymbol{a}}{\boldsymbol{l}}}$ (cm^3^)	${\boldsymbol{G}}{\boldsymbol{T}}{{\boldsymbol{V}}}_{{\boldsymbol{a}}{\boldsymbol{u}}{\boldsymbol{t}}{\boldsymbol{o}}}$ (cm^3^)	${\boldsymbol{\Delta }}{\boldsymbol{V}}({\boldsymbol{ \% }})$
1	196.20	234.63	19.59
2	37.59	33.51	10.85
3	199.33	216.71	8.72
4	131.40	140.70	7.08
5	157.12	134.47	14.42
6	194.37	189.01	2.76
7	119.63	111.62	6.70
8	155.97	145.91	6.45
9	145.98	143.55	1.66

GTV = gross tumour volume; ${\rm{\Delta }}V$ = volume difference between $GT{V}_{auto}$ and $GT{V}_{manual}$.

Table 2 shows the evaluation results of DSC, sensitivity and specificity. For all 9 cases, DSC values are higher than 0.80, ranging from 0.84 to 0.92. The mean DSC value is 0.88 (±0.02), revealing that there is a high concordance between $GT{V}_{auto}$ and $GT{V}_{manual}$ in volume and shape. The sensitivity of auto-segmentation is over 0.80 in all patients, ranging from 0.81 to 0.92 with mean value of 0.87 (±0.04). And the specificity is higher than 0.95 in all cases and its mean value is 0.98 (±0.01) with the highest value of 0.99 (patient No. 2, 5, 7, 8 and 9). The high sensitivity and specificity values demonstrate that the auto-segmentation based on the fuzzy feature fusion method shows good performance compared with the manually defined ground truth.

**Table 2:** Evaluation of the auto-segmentation results in terms of DSC, sensitivity and specificity for each patient.

Patient	DSC	Sensitivity	Specificity
1	0.84	0.92	0.95
2	0.87	0.82	0.99
3	0.87	0.91	0.97
4	0.87	0.90	0.98
5	0.87	0.81	0.99
6	0.89	0.88	0.98
7	0.87	0.84	0.99
8	0.90	0.87	0.99
9	0.92	0.91	0.99
Mean ± SD	0.88 ± 0.02	0.87 ± 0.04	0.98 ± 0.01

DSC = Dice’s similarity coefficient; SD = standard deviation.

**Figure 6:**
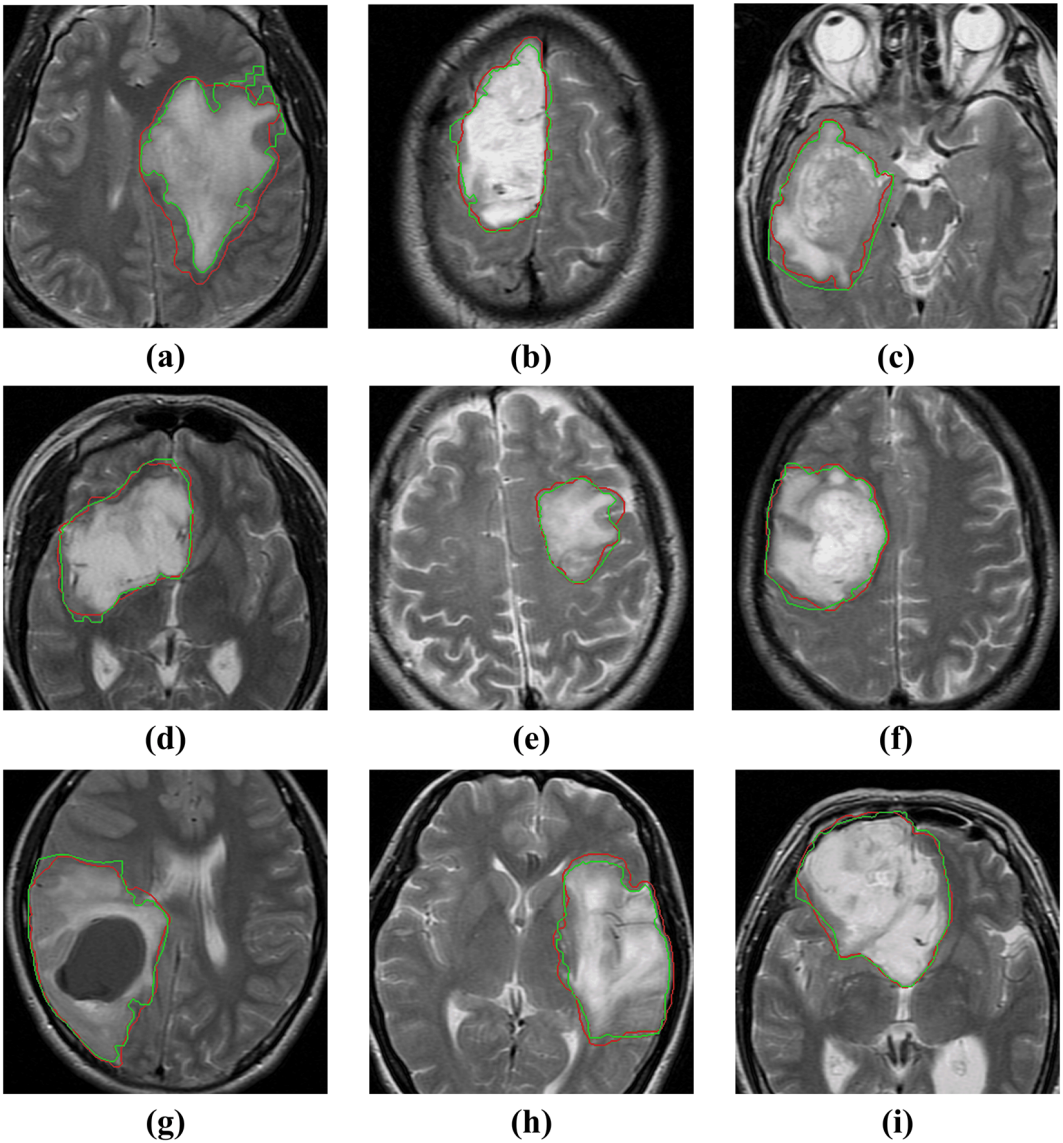
Comparison between manually delineated gross tumour volume (GTV) and automatically segmented GTV on axial slices of T2-weighted MRI images for 9 patients. (**a**–**i**) Represent patient No. 1~9 respectively.

High concordances between the contours of $GT{V}_{auto}$ and $GT{V}_{manual}$ are also observed on axial slices of T2 images for all 9 patients as shown in Fig. 6. In spite of the variance of tumour in size, location, shape for different patient, the contours of $GT{V}_{auto}$ cover most tumour area and match to $GT{V}_{manual}$ at a large degree.

## Discussion

Our study has shown that the new fuzzy feature fusion method provides an effective and reliable way to use multi-modality diffusion and perfusion MR images in radiotherapy treatment planning of gliomas. The volumes and shape of GTV defined with this new method are satisfying for all 9 patients compared with the ground truth. Moreover, the mean sensitivity and specificity of the auto-segmentation is 0.87 and 0.98 respectively, revealing the high efficiency and reliability of the auto-segmentation frame work based on the new fuzzy feature fusion method.

In the fuzzy feature extraction process, membership functions corresponding to ADC, FA and rCBV values were defined with mixed sample values from low-grade and high-grade gliomas, instead of generating membership functions for low-grade and high-grade gliomas respectively. Because isolated diffusion/perfusion parameters only reveal partial information about brain-tumour microenvironments and are insufficient to differentiate low-grade from high-grade gliomas. For example, ADC values provide only information about the movement of water molecules and may also be affected by various factors such as the density and composition of tumour cells, oedema, tumour necrosis, etc., which might result in inability to discriminate gliomas accurately^35^. Furthermore, FA values do not carry sufficient information for differential diagnosis of low-grade and high-grade gliomas because there may be tumours that have a higher growth potential but still do not disrupt the organization of white matter fibres in the process of their local invasion, leading to no detectable reduction in FA values^36^. For rCBV value, it is just a single parameter of tumour behaviour. In addition to tumour vascularity, criteria such as mitotic activity, necrosis and nuclear pleomorphism are important in histological grading of gliomas^37^. Thus, there is not sufficient theoretical support from reported studies for constructing membership function for low-grade and high-grade gliomas respectively. For each modality, the fuzzy model created in this study can be used to define regions with high possibility belonging to tumour and differentiate tumour region from normal tissue.

Several auto-segmentation methods based on fuzzy theory are proposed to employ multi-modality MR images for the tumour extent definition of gliomas. Based on knowledge-based technique and fuzzy segmentation, Dou *et al*. presented a fuzzy information fusion frame work and an average probability of correct detection 96% and an average probability of false detection 5% were obtained through studies of T1-weighted, T2-weighted and proton density images of four patients^9^. This perception-based method can generate fuzzy membership function with the prior knowledge about tumours described by experts, but lacks a general principle such as maximum likelihood to estimate probability density of input features^21^. Compared with this perception-based method, our study explored a histogram-based method to the generation of membership functions, which can provide information regarding the probability distribution of parametric values. Furthermore, the fusion method proposed by Dou *et al*. aimed at combination of structural MR images, which may not be suitable for functional multi-parametric MR images. Recently, FCM algorithm was used in the semi-automatic segmentation method to detect different regions of glioblastoma with the combination of information provided by T2 images, ADC and rCBV maps in a study proposed by Fathi Kazerooni *et al*.^16^. The results show over 80% sensitivity, specificity and dice score in the differentiation of various tumorous regions. However, the shapes of the fuzzy membership functions would be affected by the presence of noise in parametric MR maps^21^. Thus, pre-processing is an important step to deal with image noise before segmentation because the parametric maps are often with low resolution and high noise due to the long-time acquisition and calculation process. Different from this study, our new fuzzy fusion method employs membership functions to interpret parametric values with possibility belonging to tumour, instead of just treating them as image intensity and segmenting into separate clusters. As the results of evaluation indicate, the proposed method yields more than 0.85 of mean DSC, sensitivity and specificity in segmentation of gliomas, which is higher than the study mentioned above.

## Conclusions

High accuracy in automatic delineation can be achieved with the proposed method which shows promising potential of utilizing functional multi-parametric images for GTV definition in precision radiation treatment planning of gliomas.
